# primerJinn – a tool for rationally designing multiplex PCR primer sets and in silico PCR

**DOI:** 10.21203/rs.3.rs-3025970/v1

**Published:** 2023-06-26

**Authors:** Jason D Limberis, John Z Metcalfe

**Affiliations:** University of California, San Francisco

## Abstract

**Background:**

Multiplex PCR amplifies numerous targets in a single tube reaction and is essential in molecular biology and clinical diagnostics. One of its most important applications is in the targeted sequencing of pathogens. Despite this importance, few tools are available for designing multiplex primers.

**Results:**

We developed primerJinn, a tool that designs a set of multiplex primers and allows for the in silico PCR evaluation of primer sets against numerous input genomes. We used primerJinn to create a multiplex PCR for the sequencing of drug resistance-conferring gene regions from *Mycobacterium tuberculosis*, which were then successfully sequenced.

**Conclusions:**

primerJinn provides a user-friendly, efficient, and accurate method for designing multiplex PCR primers and performing in silico PCR. It can be used for various applications in molecular biology and bioinformatics research, including the design of assays for amplifying and sequencing drug-resistance-conferring regions in important pathogens.

## Introduction

Multiplex PCR amplifies numerous targets in a single tube reaction and is essential in molecular biology and clinical diagnostics. One of its most important applications is in the targeted sequencing of pathogens. By using multiple primer pairs to amplify specific target regions in a single reaction, multiplex PCR allows for the simultaneous detection and identification of multiple pathogens or drug resistance-conferring regions in a single sample, making it a valuable tool for diagnostic and epidemiological studies. Despite this importance, few tools are available for designing multiplex PCR primers,^1–6^ some of which are no longer accessible. This is because designing primers for multiple targets simultaneously is challenging, requiring careful consideration of multiple factors such as primer specificity, amplicon length, and primer interactions (dimer formation) under the specific reaction conditions. Yet, these parameters are critical for designing efficient primer sets for use on clinical samples with low amounts of DNA.

We developed primerJinn, a tool that designs a set of multiplex PCR primers and allows for the in silico PCR to evaluate them against numerous input genomes. primerJinn uses primer3^7^ to create primers and a clustering method to select the best primer set based on the amplicon size, melting temperature, and primer interactions. The in silico PCR function uses blast^8^ to identify primer pairs that amplify a DNA sequence of a user-specified maximum, at a given annealing temperature and salt concentration and provides detailed information about the primers and amplicons. Our tool also incorporates approximations for melting temperatures utilizing Q5 Hot Start High-Fidelity Polymerase buffers (NEB, USA), which differ significantly from most other polymerases. primerJinn provides an efficient and accurate method for designing multiplex PCR primers and performing in silico PCR and can be a valuable resource for researchers in the field of targeted sequencing for pathogens.

## Implementation

primerJinn uses primer3 to design primers for each specific target range in an input fasta file. By default, these primers amplify a region of 400–800 nucleotides, have an optimal length of 20 (range 10, 40) nucleotides, an optimal Tm of 65°C (range 62°C, 68°C), and are specific to the input template if a mispriming fasta is provided. In addition, the portion of the fasta file provided that is not used to design a particular primer pair is appended to the mispriming library. Since high-fidelity polymerase buffers tend to increase the Tm of primers, we have included an approximation for the highest-fidelity polymerase, Q5 from NEB. We also use the NEB Tm calculator API to output the final Tm for the selected primer set. Following the design of one hundred (default value) primer pairs for each region, a matrix is constructed including Tm, amplicon size, and heterodimer formation probability (based on the Gibbs free energy) and used to generate clusters using a Euclidean metric and Ward linkage criterion. The cluster with the most regions covered is selected, and missing primers are added from the next closest cluster. The output is written to an Excel file.

primerJinn also allows for the in silico PCR evaluation of primers. It takes a reference fasta file and primer sequences as input and returns the binding position (located using blastn-short) and product length of any pair of primers that generate a product at, or below the input Tm (default is 70°C) and the maximum amplicon size (default is 2000 nucleotides). Options include annealing temperature, salt concentration, maximum product length, and whether two or more bases at 5’ end of the primer are required to bind. The output is written to an Excel file.

## Results

**Figure 1:**
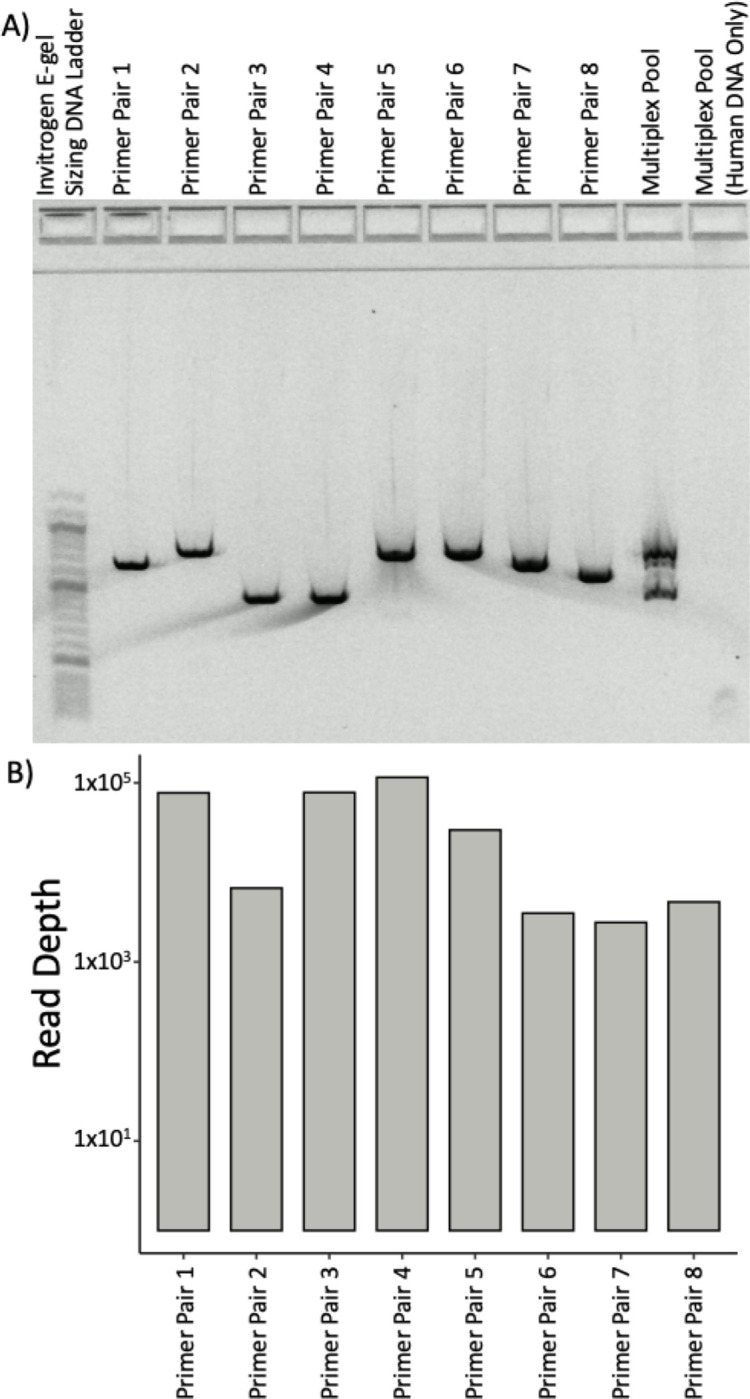
A) Electrophoresis gel showing expected amplicon sizes and specificity for each individual and pooled primer pairs and B) coverage of the sequencing of the pooled multiplex PCR from lane 8.

To evaluate primerJinn we selected eight drug resistance-conferring gene regions from *Mycobacterium tuberculosis*, the etiological agent of tuberculosis. We passed the 4.4Mb, high GC genome fasta (NC_000962.3) to primerJinn with the regions listed in Table 1. primerJinn output one primer set for each position (Table 2), with the mean primer Tm and amplicon size being 65°C (range 64°C, 67°C) and 665 nucleotides (range 454, 791), respectively. We then used these primers as input for primerJinn in silico PCR function, which appropriately returned only the eight expected amplicons. Finally, we synthesized the 16 primers and performed singleplex and multiplex PCR on *M. tuberculosis* H37Rv genomic DNA using NEB Q5 HotStart DNA polymerase MasterMix for 35 cycles with denaturation, annealing, and extension for 10s at 98C, 20s at 65C and 30s at 72C. We ran electrophoresis gels and saw the expected bands (Fig. 1A), which showed no mispriming to the human genome (lane 10). We then sequenced the amplicon pool and saw only the expected sequences (Fig. 1B). We also evaluated our Q5 Tm approximation settings against 10000 random DNA sequences from 15 to 25 nucleotides long (1000 in each group). Our Tm approximations were a mean of −0.21°C (SD 0.71°C) below that of the NEB Tm.

## Conclusion

primerJinn provides a user-friendly, efficient, and accurate method for designing multiplex PCR primers and performing in silico PCR. It can be used for various applications in molecular biology and bioinformatics research, including the design of assays for amplifying and sequencing drug-resistant conferring regions in important pathogens.

## Figures and Tables

**Table 1:** Genomic targets for primerJinn multiplex design of drug resistance-conferring regions on *M. tuberculosis* H37Rv (NC_000962.3).

Gene	Drug	Genomic position start	Genomic position end
*gyB*	Fluoroquinolones	6578	7250
*fgd1*	Delaminid	490900	491416
*rpoB*	Rifampicin	761007	761277
*rv0678*	Bedaquiline	778990	779488
*fbiC*	Linezolid	1303831	1303911
*atpE*	Bedaquiline	1461045	1461291
*inhA*	Isoniazid	1674182	1674222
*katG*	Isoniazid	2154831	2154873
*pncA*	Pyrazinamide	2288681	2289242

**Table 2:** primerJinn output for eight drug resistance conferring gene regions of M. tuberculosis H37Rv (NC_000962.3). Annealing temperatures (Tms) are in Celsius (Tms have been rounded for illustration purposes), and the product sizes in nucleotides.

Forward Primer	Reverse Primer	Forward Tm	Reverse Tm	Product Size	Name	Forward Primer Tm NEB	Reverse Primer Tm NEB
GAGGAACACCACTAGTACCG	CTCGATGACTTTACGGCCAT	65	64	675	Target 1461045–1461291	64	64
CATGGGATATGGAGCGATCG	GGGGTCGTAGGAGATCTTGA	66	66	791	Target 490900–491416	65	65
CCGGTTGTCCATTCCGTTTA	CTGTACGTATTTGGGTTGCG	64	66	454	Target 1303831–1303911	65	64
GGATGCGAGCTATATCTCCG	AATACGCCGAGAT GTGGAT G	65	65	458	Target 1674182–1674222	65	65
CAACAGTTCATCCCGGTTCG	GACGGATTTGTCGCTCACTA	65	67	759	Target 2288681–2289242	66	64
GCCACCATCGAATATCTGGT	GCTCCAGGAAGGGAATCATC	66	65	778	Target 761007–761277	64	65
GCATACCGAACGTCACAGAT	ACGGTCACCTACAAAAACGG	66	65	665	Target 778990–779488	65	65
GCTCTTAAGGCTGGCAATCT	CGGTCACACTTTCGGTAAGA	65	66	577	Target 2154831–2154873	65	64

## Data Availability

Code and raw data are available at https://github.com/SemiQuant/PrimerJinn.
